# The Tasmania-London protocol to detect isolated rapid eye movement sleep behavior disorder using home-based video-polysomnography

**DOI:** 10.1093/sleep/zsaf313

**Published:** 2025-10-07

**Authors:** Samantha Bramich, Alastair J Noyce, Anna E King, Séan Higgins, Cristina Simonet, Aidan D Bindoff, Sharon L Naismith, James C Vickers, Laura Pérez-Carbonell, Jane Alty

**Affiliations:** Wicking Dementia Research and Education Centre, University of Tasmania, Hobart, Australia; Wicking Dementia Research and Education Centre, University of Tasmania, Hobart, Australia; Centre for Preventive Neurology, Wolfson Institute of Population Health, Queen Mary University of London, London, UK; Wicking Dementia Research and Education Centre, University of Tasmania, Hobart, Australia; Sleep Disorders Centre, Guy's and St Thomas' NHS Foundation Trust, London, UK; Wicking Dementia Research and Education Centre, University of Tasmania, Hobart, Australia; Centre for Preventive Neurology, Wolfson Institute of Population Health, Queen Mary University of London, London, UK; Wicking Dementia Research and Education Centre, University of Tasmania, Hobart, Australia; School of Psychology, Brain and Mind Centre, University of Sydney, Sydney, Australia; Wicking Dementia Research and Education Centre, University of Tasmania, Hobart, Australia; Centre for Preventive Neurology, Wolfson Institute of Population Health, Queen Mary University of London, London, UK; Sleep Disorders Centre, Guy's and St Thomas' NHS Foundation Trust, London, UK; Institute of Psychiatry, Psychology and Neuroscience, King’s College London, London, UK; Wicking Dementia Research and Education Centre, University of Tasmania, Hobart, Australia; School of Medicine, University of Tasmania, Hobart, Australia; Neurology Department, Royal Hobart Hospital, Hobart, Australia

**Keywords:** neurodegenerative diseases, parasomnias, diagnosis, ISLAND

## Abstract

**Study Objectives:**

Isolated rapid eye movement (REM) sleep behavior disorder (iRBD) is an early manifestation of alpha-synuclein-related neurodegenerative diseases (NDD). There is an average delay in iRBD diagnosis of 9 years from the onset of symptoms, showing that we need easier methods of detection to improve access to specialist care and potential disease-modifying clinical trials. We designed a 3-step approach to detect iRBD in a community of older adults (≥50 years) in Tasmania, Australia, using home-based video-polysomnography (vPSG).

**Methods:**

The Tasmania-London (TASLON) protocol for iRBD detection comprised 3 key steps: participants completed an online iRBD screening question; those who screened positive were invited to undertake the TASLON iRBD Screening Interview by telephone; a sample then completed a home-based vPSG based on iRBD screening risk level.

**Results:**

A total of 2891 participants (mean [SD] age 64 [7.7] years; 74% female) without any known NDD were recruited from throughout Tasmania. 267 (9%; age 63 [7.7] years; 55% female) were identified as having “probable” RBD through positive online screening; 87 (33%) agreed to complete the clinical screening interview; 47 (55%) underwent home-based vPSG; and 21 (45%; age 68 [7] years; 52% female) were found to have iRBD.

**Conclusions:**

The TASLON protocol is a feasible method of improving timely access to iRBD diagnoses in the community. It streamlines the path to vPSG by identifying those at highest risk of iRBD, thus improving access to diagnostic testing and clinical trial opportunities for those who otherwise may not have been identified.

**Statement of Significance**

The TASLON protocol offers a novel, community-based approach to early detection of isolated REM sleep behavior disorder (iRBD), a precursor to alpha-synuclein-related neurodegenerative diseases (NDD). By streamlining the path to home-based video-polysomnography (vPSG), bypassing the need for hospital/laboratory-based vPSG, this method addresses a critical delay in iRBD diagnosis, especially for populations with limited access to specialist sleep centers. This study highlights the feasibility and preliminary results of the TASLON protocol in detecting new cases of iRBD in the community. This holds potential to reduce inequity of access to early interventions and may improve patient outcomes in NDD through enhanced access to specialist care and clinical trials. Future work should focus on extending this approach and exploring its applicability in broader populations to aid access to earlier diagnosis.

## Introduction

Isolated rapid eye movement (REM) sleep behavior disorder (iRBD) is a parasomnia that is characterized by a loss of normal atonia during REM sleep, which often results in dream enactment [1]. It is an early manifestation of alpha-synuclein-related neurodegenerative disease (NDD), including Parkinson’s disease (PD), dementia with Lewy bodies (DLB), and multiple system atrophy (MSA) [2]. Identification of people with iRBD is a key research priority worldwide as approximately 80% will progress to an NDD within 14 years [3]. Early detection offers a critical opportunity to intervene with disease-modifying clinical and pharmaceutical trials to potentially delay or even prevent the onset of NDD.

However, it can be challenging to detect iRBD in the community and it is vastly underdiagnosed [4, 5]. Currently, diagnosis is made according to criteria detailed in the International Classification of Sleep Disorders, Third Edition (Text Revision; ICSD-3-TR) 2023 (published by the American Academy of Sleep Medicine [AASM]); this states that evidence of REM sleep without atonia (RSWA; loss of muscle atonia in REM sleep) needs to be confirmed via polysomnography (PSG) (a specialist sleep study) together with a convincing clinical history of dream enactment behavior [6]. This approach is problematic due to limited access to PSG and sleep specialist services, especially in rural and remote parts of the world [7, 8]. Hence, those with iRBD may go undiagnosed for years before overt symptoms of a NDD appear. This is a clear barrier to treatment and intervention, with recent studies showing that a substantial number of people with iRBD go on to develop either DLB (50% of cases), PD (45% of cases), or MSA (5% of cases) [9].

Alongside PSG, clinical interview is used to gather details of dream enactment behavior based on the ICSD-3-TR criteria. This relies on an in-depth understanding of the symptoms and presentation of iRBD and potential mimics of the disorder. Whilst a small number of specialist clinicians, such as neurologists who specialize in neurodegenerative disorders and sleep specialists/physicians, will have knowledge of iRBD, most medical training programs do not provide detailed education on the intricacies of sleep, still less on sleep disorders like iRBD [10–12]. As such, clinical interviews vary in consistency due to a lack of formal knowledge or terminological ambiguity [11, 13]. Unfortunately, no standardized clinical iRBD interview exists to aid clinicians in these assessments.

Several validated screening questionnaires are available to assist, and these are usually short and succinct, but vary in accuracy, with stated sensitivity values ranging from 64% to 100% and specificity between 36% and 100% [14]. When compared to gold-standard PSG, all have been found to show low specificity estimates and low positive predictive values [15]. This is likely due to their inability to rule out other sleep disturbances and disorders that can mimic iRBD symptoms, such as obstructive sleep apnea (OSA), non-REM (NREM) parasomnias, and severe periodic limb movement disorder (PLMD) [7]. One of the most commonly used screening questions is the REM Sleep Behavior Disorder Single Question Screen (RBD1Q) that simply asks, “Have you ever been told, or suspected yourself, that you seem to ‘act out your dreams’ while asleep (for example, punching, flailing your arms in the air, making running movements, etc.)?” [16]. It has been shown to have a sensitivity of 80% and specificity of 75.3% for PSG-confirmed iRBD [14] making it one of the most reliable screening measures for iRBD currently available [7]. However, as outlined by Stefani et al, clinical diagnoses cannot be made on screening questionnaires alone as the specificity and positive predictive values are too low [15].

In response to this urgent need for more accessible pathways to iRBD diagnosis, the Tasmania-London (TASLON) protocol was proposed as a pragmatic approach to overcome some of these “real-world” challenges to iRBD detection. The aim of this protocol paper is to describe the TASLON protocol, which uses a combination of screening questionnaire, a semi-structured clinical interview, and a home-based video-recorded PSG (vPSG). For Tasmania, an island state in Australia, there is a lack of accessibility to sleep specialists and sleep investigations, and this protocol was developed with these challenges in mind. The Tasmanian ISLAND Sleep Study is an ongoing project that aims to characterize iRBD, and the detailed protocol has previously been published [17]. Here, we outline the TASLON protocol and preliminary results from the ISLAND Sleep Study cohort. We hypothesize that, by using this approach, we will accurately identify a cohort of people with iRBD in Tasmania, Australia. We envisage that this will then provide future researchers and clinicians with a valuable protocol to detect iRBD more easily, efficiently, and with less expense.

## Materials and Methods

### Ethics

This study has been approved by University of Tasmania’s Health and Medical Human Research Ethics Committee (HREC 26435 and HREC 18264) and was conducted in accordance with the National Health and Medical Research Council’s National Statement on Ethical Conduct in Human Research (2018). Participants were given up-to-date information on iRBD, its relevance to NDD, and the overall aims of the project [17]. They were asked to provide consent before completing the online questionnaires, telephone screening interview, and home-based assessments.

### Study population

Participants were recruited from the general population of Tasmania, Australia. Eligibility criteria were (1) being a resident in Tasmania, (2) being aged 50 years or older, and (3) being a participant in the Island Study Linking Aging and Neurodegenerative Disease (ISLAND) Project. The ISLAND Project is a 10-year public health initiative launched in 2019 by the Wicking Dementia Research and Education Centre, aiming to build dementia risk management self-efficacy and decrease dementia risk in Tasmanians aged 50 or older [18].

All participants in this project enrolled in the Tasmanian ISLAND Sleep Study, which is a substudy of the ISLAND Project; inclusion criteria and recruitment strategies for the ISLAND Project [18] and the Sleep substudy have been previously published [17]. In short, this substudy is a longitudinal, prospective observational study investigating iRBD in Tasmania. It aims to determine the population prevalence of iRBD in adults aged ≥50 years, explore iRBD characteristics and biological markers to determine profiles that are specific to people with iRBD and investigate their contributions to alpha-synuclein NDD phenoconversion. Alongside this, all participants are encouraged to take part in the ISLAND project’s strategies to maximize brain health in line with *Lancet Commission on dementia prevention* recommendations [19]. This is done by providing free online Understanding and Preventing Dementia MOOC’s (Massive Open Online Courses; health promotion education), personalized feedback detailing modifiable risk factors, monthly newsletters on healthy aging, webinars, and a number of community events [18].

### Study procedures

The TASLON protocol to detect vPSG confirmed iRBD in the community comprises three steps as follows:

*Step 1.* Participants completed a battery of online validated questionnaires (see Supplementary Table S1), including the RBD1Q [16] to screen for “probable” RBD (pRBD) status.

*Step 2.* Prior to consenting to participation in the next steps of the study, participants were sent detailed information about the project and the relevance of an iRBD diagnosis. Mindful of the ethical issues around disclosing a new diagnosis of iRBD, participants were also required to consent to the research team informing both the participant and their general practitioner (GP) of results. Participants were free to decline participation if they did not want to be informed of a potential iRBD diagnosis. Diagnostic disclosure was an ethical requirement of the University of Tasmania’s Health and Medical Human Research Ethics Committee.

Consenting participants who screened positive on the RBD1Q (pRBD) were invited to undertake a 15-question telephone screening interview (with their bed partner, if applicable) to ascertain further details about iRBD symptoms (see Table 1). This was constructed based on established iRBD symptomatology [1] in collaboration with experts in the field of neurology and sleep medicine (LPC, AJN, CS, SB, JA). Specifically, questions 5, 6, and 7 were replicated from the Oxford-Luxembourg collaboration with permission (see acknowledgements).

Participants were contacted by a research team member (SB), who is a qualified sleep scientist and asked each interview question remotely, over the telephone. If a bed partner was available, they were included in a conference call or phoned separately, and answers were combined with those of the participant. Interviews took between 20 and 40 minutes to complete, depending on the depth of detail available. Questions were asked in the order presented in Table 1 and participants and bed partners were free to provide additional details at any time throughout the interview process. All answers were written down verbatim throughout the interview and then transcribed into a secure database.

The researcher, advised by the sleep medicine specialist, senior author/LPC (who was blinded to any iRBD screening question results), evaluated participants’ answers to interview questions based on keywords or phrases, and determined those who were most likely to have symptoms consistent with iRBD. Answers were categorized into three groups based on risk for iRBD: high, medium, and low (see Table 2). The “high-risk” and “medium-risk” subgroups were then invited to undertake a home-based vPSG to detect true iRBD.

Examples of key “high-risk” participant interview responses included: “Yes, I have fallen out of bed and knocked things off the bedside table”; “I often dream of running away or trying to defend myself or others”; and “I have never sleepwalked, but I have stood up in my sleep, maybe twice.” Key bed partner responses included: “He does frequently kick and punch in his sleep, at least once per week”; “He does lash out and hit me. He hit me harder than normal recently and kicked me quite hard”; and “She has gotten out of bed a few times, still asleep, and fallen over. She has hit her head on the bedside table before.”

*Step 3.* Over a 12-month period, the home-based vPSG was conducted in the participant’s home or, by request, in a hotel room. The researcher (SB) or a trained research assistant (RA) who had healthcare experience and was familiar with the medical equipment and building rapport with patients, performed the vPSG set-up for each participant. The staff placed all the electrodes before they left the participant’s home, usually in the early evening, to obtain data for one night of sleep, and then they returned the next morning to detach all the equipment. The RA was trained to perform a full vPSG set-up to meet AASM standards (please see below details on montage used) [6]. The researcher demonstrated the home-based vPSG set-up to the RA three times, provided two direct-observation supervision sessions to ascertain the RA had gained the required skills, after which the RA undertook study set-up independently. The researcher and RA met intermittently to set-up home-based vPSGs together to ensure accurate application of all sensors was ongoing.

**Figure 1:**
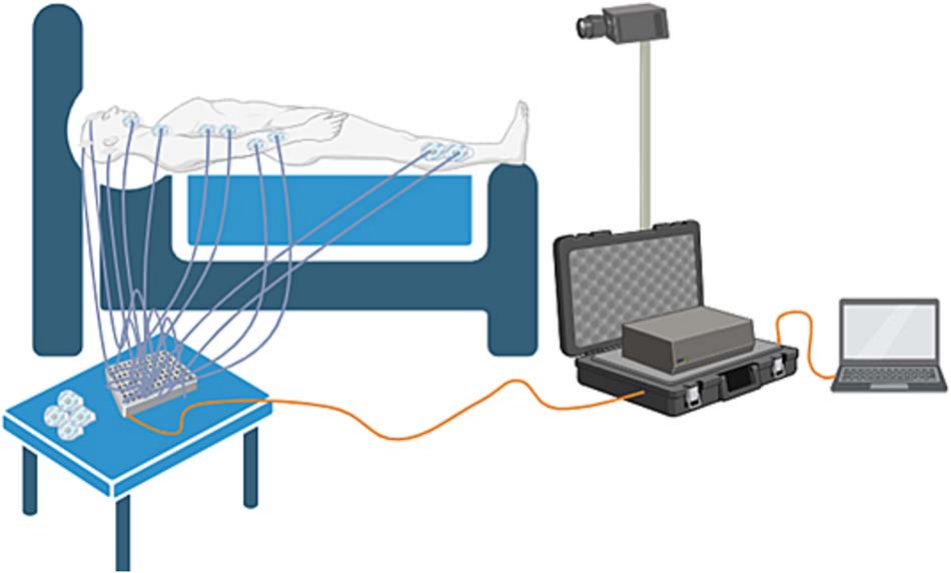
Example of home-based vPSG bedroom set-up. Depicts Grael headbox placed near head of the bed connected to the ONsight system by data cable; camera (attached to ONsight system) placed at the foot of the bed for clear view of the participant; ONsight system connected to screen via power cable; not shown—ONsight system powered via connection to electrical socket in the home.

The home-based vPSG equipment for this study was configured in collaboration with Compumedics Australia, a commercial company that develops, manufactures, and commercializes diagnostic technologies (https://www.compumedics.com.au/en/). This system was chosen because it was ambulatory and could be configured to include all data acquisition needed to detect iRBD using a Level II-type study [40]. It consisted of an ONsight A.V.S. recording computer, configured to a Grael 4K PSG amplifier, which is currently used in several hospital-based sleep laboratories throughout Australia. This allowed for the collection and recording of all physiological data required to determine the presence of iRBD, or other sleep disorders, in line with the AASM Manual for the Scoring of Sleep and Associated Events [6]. Data collected included: 10 EEG channels (using the 10–20 system; F3, F4, C3, C4, O1, O2, A1, A2, reference and ground), electrocardiogram, electrooculogram, thoracic and abdominal respiratory effort, nasal airflow, oximetry, EMG assessment of the following muscles: submentalis (chin), bilateral flexor digitorum superficialis (arms), bilateral anterior tibialis (legs), and synchronized video and audio recording. If participants had a previous diagnosis of OSA and were on continuous positive airway pressure (CPAP) therapy, this was used on the night of the study as usual. The researcher or RA transported the vPSG equipment to the participant’s home (by standard car) and positioned the Grael headbox next to the patient’s bed (usually on a bedside table) and the ONSight system at the foot of the bed (see Figure 1).

**Table 1:** TASLON iRBD screening interview questions with inclusion rationale

**Introduction** Thank you for speaking with me. We are calling because we wish to clarify some of the questionnaires that you filled in as part of the ISLAND Sleep Study. We are especially focusing on sleep problems. Based on your answers in the online survey, it seemed like you may have REM sleep behavior disorder. Let me explain what this is and let’s decide together if you really might be affected by this sleep disorder. REM sleep behavior disorder is a sleep problem in which people “act out their dreams” at night. Normally, when we dream in REM sleep, we are paralyzed, but in this disorder, we are not; so whatever we are dreaming about, we can do. When the disorder is mild, people may just talk—generally this is more than a brief mumbled phrase—often one may appear to be carrying on a conversation. Sometimes with this there will be laughing or crying. People may also move—for example, they may reach for imaginary objects or make running movements in bed. If the disorder is severe, the movements can be dangerous, such as punching or kicking, or throwing oneself out of bed. Usually, it looks like the person is acting out a dream, and often, if they are woken, people might say that their behavior was matching the content of their dream.
**Questions**
1. Do you suspect that you “act out your dreams” while asleep (for example, movements such as punching, flailing your arms in the air, kicking, shouting, swearing, laughing in your sleep, etc.) *Rationale: The RBD1Q is used to confirm original response to the online question* [16]*.*
2. Do you have frequent vivid dreams, often of a distressing content (e.g. being attacked, needing to defend yourself from a threat)? *Rationale: Vivid and distressing dreams are commonly reported by people with iRBD* [20]*.*
3. Do you have a bedpartner/someone who sleeps in the same bed/room as you sometimes? (a) if yes, have they told you that you seem to act out your dreams? *Rationale: Bed partner interview is known to increase the sensitivity of the RBD1Q* [15]*.*
4. Can you tell me about a time when you thought or were told that you acted out your dreams whilst asleep? how many times has this happened? approximately what time of night did this/do these occur (first half/second half)? at what age did you have one of these episodes for the first time? have you ever walked around the bedroom or gone to other areas of the house during one of these episodes? how severe were the movements/did you injure yourself or your bedpartner? have you fallen out of bed as a consequence of episodes where you were acting out your dreams? have you ever spoken to a doctor about these nighttime movements? *Rationale: Clinical history of repeated episodes of sleep-related vocalization and/or complex motor behaviors is an ICSD-3-TR requirement for iRBD diagnosis* [6]
5. There are a few other explanations for movements that can happen during the night other than REM sleep behavior disorder. One is sleepwalking and sleep talking. This often starts when you are younger (childhood or adolescence). During an episode, it seems like the person is half asleep and half awake. With this, people can talk, walk around, etc. There are some clues that can help us distinguish this, which I will ask you about: during the episodes, might you walk? can you interact with someone during the episode, or does interaction happen only after you are woken? For example, if someone will talk to you during an episode, you might reply (while still in the episode)? other than grabbing or hitting something in the immediate vicinity, might you interact with the environment during episodes (for example, reaching out to take an object, drinking from a glass of water, brushing teeth with a toothbrush, opening a door)? are these episodes what you were referring to when you answered yes to the first question, or are there other movements as well? *Rationale: NREM parasomnias, such as sleepwalking, can mimic iRBD symptoms. Agreement to questions in this section may explain sleep-related motor behavior other than iRBD* [21]*, however, sleep talking can also be a feature of iRBD* [22]*. Additionally, adult-onset episodes of wandering around in sleep may also be seen in association with iRBD* [23]*.*
6. Another frequent sleep problem is bad snoring and sleep apnea. Do you know if you have sleep apnea or snore? Bad snoring episodes can also cause movements sometimes. Do you snore loudly enough to be heard in the next room? Do most of the movements seem to occur related to snoring? *Rationale: Snoring and sleep apnea can also cause movements in sleep. Agreement to this section may explain sleep-related motor behavior other than iRBD, however, sleep apnea can occur alongside iRBD* [21, 24, 25]*.*
7. There are some other simple movements that people might do while sleeping that are not acting out dreams. These include generalized body jerks especially when falling asleep, or a need to move the legs or arms before falling asleep, or rhythmic movements in the legs while asleep. Do these happen to you? *Rationale: Hypnic jerks, sleep-related head jerks or severe PLMD may account for some described movements* [21, 26–28]*.*
8. Have you ever been diagnosed with a neurological disorder, such as PD, dementia, traumatic brain injury, stroke, etc? *Rationale: RBD can be a symptom of PD and dementia and may also result from head injury or stroke. The presence of these conditions may negate the possibility of “isolated” RBD* [29–32]*.*
9. Have you ever been diagnosed with any form of epilepsy or had a seizure/fit? *Rationale: Nocturnal epileptic seizures may account for the description of sleep-related motor behavior* [33]*.*
10. Have you ever been diagnosed with post-traumatic stress disorder? *Rationale: Patients with post-traumatic stress disorder can also experience nightmares and behaviors in sleep, with a similar clinical presentation and PSG findings to those observed in iRBD* [31]*.*
11. Have you ever been diagnosed with a sleep disorder such as obstructive or central sleep apnea, restless legs syndrome, or PLMD? *Rationale: Several sleep disorders may mimic iRBD and should be ruled out as potential explanations for the described symptoms of dream enactment* [21, 34]*.*
12. Have you ever experienced visual hallucinations (saw things that were not really there) at night? *Rationale: Visual hallucinations are a common feature of DLB and PD* [35]*. People may be undiagnosed and therefore exhibiting RBD and hallucinatory symptoms, rather than iRBD. Additionally, nocturnal hallucinations may also be reported in prodromal phases of alpha-synuclein-related NDD* [36]*.*
13. Do you have a history of sleepwalking or night terrors in childhood or adolescence? *Rationale: Sleepwalking/night terrors may have persisted throughout adult years, explaining some sleep-related motor behavior* [37]*. A childhood onset of sleep-related behaviors would point against iRBD.*
14. Do you take any regular antidepressant or sleep medication? *Rationale: There is a strong association between antidepressant use and the emergence of RBD**.** Antidepressants are known to unmask, trigger, or worsen RBD.* [38]*.*
15. Do you ever drink one or more alcoholic beverages within 2 hours of going to bed? *Rationale: Alcohol consumption can fragment sleep and has been proposed as a risk factor for the development of probable RBD* [39]*.*

**Table 2:** Examples of high, medium and low-risk iRBD interview responses

**High Risk**	**Q. #**	**Response**
	1	Yes, I do this
	2	Yes, I often dream of running away or trying to defend myself or others
	3	Yes, my husband notices regular dream enactment behavior, always in the 2nd half of night, usually around 3–4 am.
	4	My husband often sees me reaching out with my arms and I remember dreaming of rescuing children (I am an ex-school teacher). I think it started when I was in my late 50s, after a high work stress situation. Husband reports: it happens 3–4 times/week. Sometimes she might go a fortnight without anything but then every night for a week. I need to wake her up fully for it to stop, otherwise she goes straight back to sleep and continues moving. I sleep with my hands in front of my face to prevent being punched. She rarely gets out of bed, has done it maybe 2–3 times, but her arm movements are frequent, kicking not as often but it does happen.
	5	I’ve never sleepwalked but have stood up maybe twice. Husband: She sleep talks regularly though—like full conversations.
	6	Husband: Occasionally she snores, not very often, but always in the first part of the night.
	7	No, not that I can remember.
	8	No
	9	No
	10	No
	11	No
	12	No
	13	No
	14	No
	15	No
**Medium Risk**	**Q.#**	**Response**
	1	Yes, I think I have had dreams where I’ve kicked out and I’ve been told I sleep talk a lot
	2	Not frequently distressing, but frequently vivid.
	3	No, but I was holidaying with a friend recently and she reported a lot of sleep talking. Years ago, when I was younger someone also told me I was yelling in my sleep when I stayed in a youth hostel.
	4	3 years ago, I was dreaming about kicking a monster and I kicked my toe into the bookshelf and broke it. Last night I was yelling in my dream and woke myself up. I think I move my hands around a lot as often things on the bookshelf next to my bed have fallen off when I wake up. These episodes are not regular, I think I’ve had maybe a dozen that I know of. I have kicked my cats across the room when dreaming before but I don’t think I’ve ever gotten out of bed. I think they happen in the 2nd half of the night. I cannot remember when they first started as an adult, but I do remember sleepwalking as a child and I strongly recall vivid dreams in my 30s. I haven’t talked to my doctor about it.
	5	Yes sleep talking is often reported whenever I share a room with friends. I have a history of sleepwalking when I was young but I don’t think I’ve done it as an adult.
	6	I don’t know if I snore, but my friend did not report snoring when we were away together.
	7	Yes, hypnic jerks are fairly common but different to these other movements.
	8	No, but I have wondered if I had a TIA <transient ischaemic attack> 10 years ago, it was never diagnosed though.
	9	No
	10	No
	11	No
	12	No
	13	Yes, I was told I sleepwalked to my nan’s house next door when I was a child, and I remember possibly falling out of a bunk bed, but not 100% sure if that was me or my sister.
	14	Yes, I take an antidepressant (escitalopram)
	15	Sometimes, but not regularly and not in the last year.
**Low Risk**	**Q.#**	**Response**
	1	Maybe, I did have a home sleep study done recently though and it found severe OSA. Since I started CPAP my sleep has been much better.
	2	Yes, I have always had nightmares and very vivid dreams. Over all my years I have done a lot of kicking and punching with my nightmares. It used to happen 3 or 4 times per year, but I haven’t noticed them since I started CPAP. My husband said I snored and moved a lot so my doctor sent me for a sleep study.
	3	Yes, my husband
	4	I used to be a sleepwalker, not for decades though. Last week I had a nightmare, and my husband said I was making distressing noises but not really moving at all. Husband: yes, her sleep is much quieter since she started CPAP.
	5	No I haven’t sleepwalked for many years. Husband: she does sleep talk sometimes though.
	6	Yes I was recently diagnosed with OSA and now use CPAP.
	7	Yes, I sometimes have hypnic jerks but not frequently. I think they are different to my other movement.
	8	No
	9	No
	10	No
	11	OSA
	12	No
	13	Yes, I sleepwalked but I don’t think I had night terrors.
	14	No
	15	No

**Figure 2:**
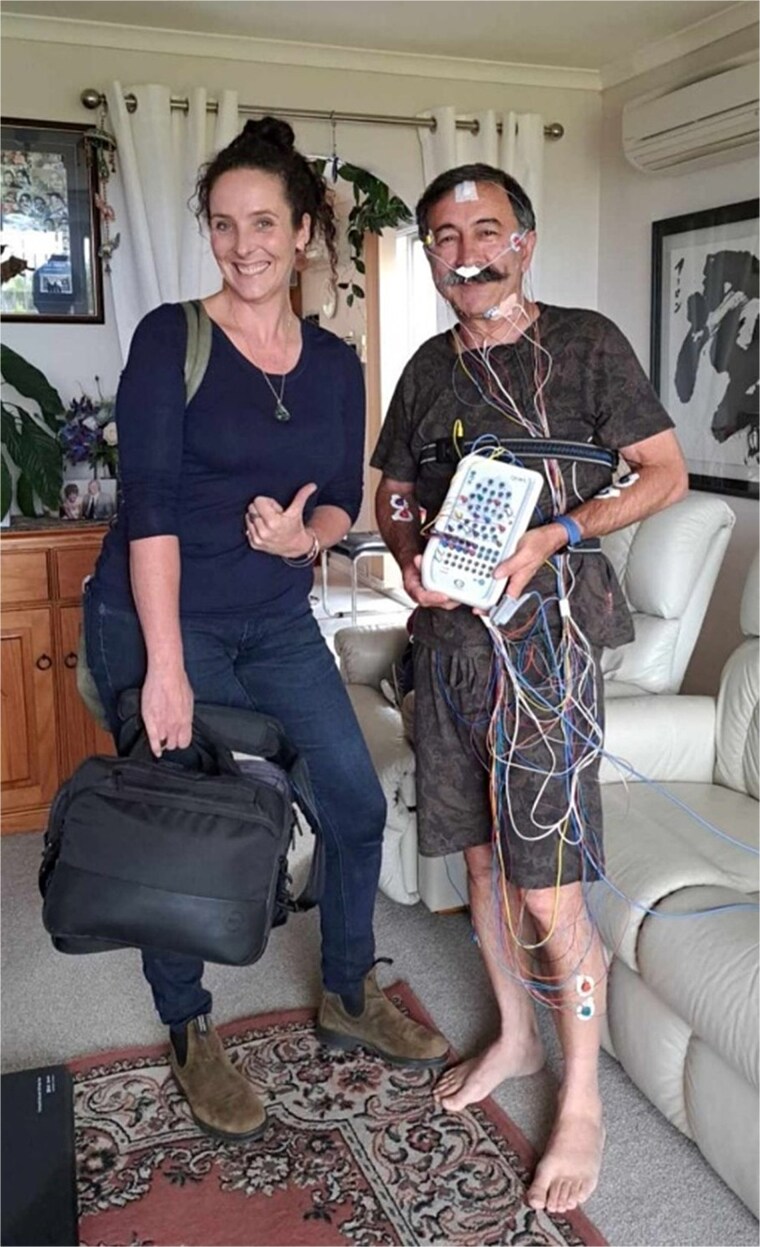
Image of RA and research participant with home-based vPSG equipment attached (with permission^*^). ^*^Written consent obtained from the RA and participant for reproduction.

Participants were informed about the need for each sensor and what data they were collecting and were encouraged to keep them intact until the following morning. The Grael headbox was able to be removed from its cradle during the evening so that the participant could carry it with them and continue with their evening activities prior to their usual sleep time, whilst also being able to use the bathroom overnight if needed (see Figure 2). As this was a preliminary study of the TASLON protocol, the researcher was available overnight by telephone to provide phone-based technical support if the participant required assistance (however, this was not utilized by any participants). The researcher or RA then returned to the home the following morning to remove the sensors, collect the equipment, and then upload the recorded data from the ONsight computer to a secure database in preparation for scoring.

### Data Analysis & Diagnosis

All vPSG data were scored in line with the AASM manual [6], first by the researcher and then by a qualified sleep physiologist with expertise in RBD detection (SH) who was blinded to all clinical data and the interview screening answers. Each participant’s scored data set was reviewed by the sleep medicine specialist (senior author/LPC; who was also blinded to all clinical data and the interview screening answers) to determine the presence of iRBD or other sleep disorders.

Those who were found to show evidence of iRBD on vPSG were notified of the results in writing and provided with information about the condition. They were offered a phone call with the researcher (SB) to discuss any concerns or queries about the results. Their preferred GP or primary care physician was also notified, and participants were invited to discuss the results with their GP. A recommendation was made for the GP to refer the participant for follow-up with the senior author/neurologist (JA) at the ISLAND Clinic, which is a one-stop interdisciplinary assessment and diagnostic clinic that incurs no out-of-pocket costs for the participants [41]. At this appointment, participants were offered further assessments including a one-hour battery of neuropsychological tests, an MRI brain scan, and careful examination, using clinical and lab-based assessments for evidence of motor, cognitive, and speech decline. Their case was discussed in an interdisciplinary consensus diagnosis meeting (attended by a geriatrician, neuropsychologist, psychologist, neurologist, and radiologist). The participants were provided with written and verbal information on iRBD, the relevance to increased risk of NDD (specifically naming PD, DLB, and MSA), known prognosis, and their treatment options for iRBD, including bedroom safety. They were invited to undertake further research assessments for future phenotyping studies and offered a continuing follow-up appointment with the neurologist every 18–24 months. In addition, a specialist letter was sent to both the participant and their GP post-clinic appointment. This details the risk associated with iRBD phenoconversion to NDD; provides advice for lifestyle risk modification to maintain good brain health; explains the current pharmacological treatment options for minimizing dream enactment behavior (i.e. melatonin and clonazepam); and recommends monitoring and modification of vascular risk factors.

## Results

A total of 2909 participants (mean [SD] age 64 [7.7] years; 74% female) were recruited from throughout Tasmania. We excluded 18 participants with a self-reported PD or Parkinsonism diagnosis. 267 (age 63 (7.6) years; 55% female) were identified as having pRBD as per the RBD1Q. Between-group differences were calculated, as shown in Table 3. A significantly greater percentage of participants in the pRBD group experienced anxiety or reported using antidepressant medications compared to controls (*p* < .001). A small but significant difference between groups was also reported for sleep quality, based on answers to the Pittsburgh Sleep Quality Index [42] (worse in the pRBD group; *p* < .05), and Aboriginal and/or Torres Strait Islander origin (First Nations people of Australia) (higher proportion in the pRBD group; *p* < .01). 

**Table 3:** Demographic and clinical data of the complete cohort

	**Control** **(*N* = 2624)**	**pRBD** **(*N* = 267)**	p^*^
**Age (in years)**			
Mean (SD)	63.9 (7.72)	62.6 (7.59)	.00943
Median [Min, Max]	64.0 [50.0, 91.0]	62.0 [50.0, 88.0]	
**Sex**			
Female	1987 (75.7%)	147 (55.1%)	<.001
Male	631 (24.0%)	119 (44.6%)	
Other	4 (0.2%)	1 (0.4%)	
Prefer not to say	2 (0.1%)	0 (0%)	
**Marital status**			
Married	1533 (58.4%)	174 (65.2%)	
Separated or divorced	353 (13.5%)	32 (12.0%)	
Defacto	274 (10.4%)	25 (9.4%)	.17
Single	206 (7.9%)	19 (7.1%)	
Widowed	171 (6.5%)	10 (3.7%)	
Other	15 (0.6%)	4 (1.5%)	
Prefer not to say	4 (0.2%)	0 (0%)	
**Highest level of education**			
Bachelor’s Degree	573 (21.8%)	43 (16.1%)	.112
Certificate or Apprenticeship (including Cert 2, 3, or 4)	258 (9.8%)	37 (13.9%)	
Diploma/Associate Degree	478 (18.2%)	50 (18.7%)	
High School	332 (12.7%)	37 (13.9%)	
Higher University degree (Honors, Graduate Diploma, Master’s or PhD)	847 (32.3%)	94 (35.2%)	
Other	84 (3.2%)	5 (1.9%)	
Primary School	1 (0.0%)	0 (0%)	
**Currently employment**			
No	1488 (56.7%)	145 (54.3%)	.329
Yes	1090 (41.5%)	121 (45.3%)	
**Currently retired**			
No	210 (8.0%)	29 (10.9%)	.0185
Yes	1312 (50.0%)	128 (47.9%)	
Unknown	0 (0%)	1 (0.4%)	
**Number of children**			
Mean (SD)	2.11 (2.44)	1.96 (1.41)	.621
Median [Min, Max]	2.00 [0, 44.0]	2.00 [0, 11.0]	
**Remoteness area^†^**			
Inner Regional Australia	1930 (73.6%)	196 (73.4%)	.335
Outer Regional Australia	664 (25.3%)	67 (25.1%)	
Remote Australia	5 (0.2%)	2 (0.7%)	
Very Remote Australia	7 (0.3%)	0 (0%)	
**Country of birth**			
Australia	1628 (62.0%)	156 (58.4%)	.0585
United Kingdom	318 (12.1%)	24 (9.0%)	
New Zealand	56 (2.1%)	3 (1.1%)	
Germany	21 (0.8%)	2 (0.7%)	
Netherlands	22 (0.8%)	2 (0.7%)	
Ireland	12 (0.5%)	1 (0.4%)	
Poland	3 (0.1%)	0 (0%)	
Philippines	1 (0.0%)	1 (0.4%)	
Italy	0 (0%)	1 (0.4%)	
Other	137 (5.2%)	22 (8.2%)	
**Aboriginal and/or Torres Strait Islander origin**			
No	2156 (82.2%)	200 (74.9%)	.0025
Yes, Aboriginal	25 (1.0%)	10 (3.7%)	
Yes, both Aboriginal and Torres Strait Islander	2 (0.1%)	0 (0%)	
Yes, Torres Strait Islander	1 (0.0%)	0 (0%)	
**Sleep quality**			
Good Sleep Quality	1101 (42.0%)	93 (34.8%)	.0223
Poor Sleep Quality	1506 (57.4%)	173 (64.8%)	
**Anxiety level**			
Abnormal	240 (9.1%)	43 (16.1%)	<.001
Borderline abnormal	403 (15.4%)	54 (20.2%)	
Normal	1945 (74.1%)	169 (63.3%)	
**Depression level**			
Abnormal	62 (2.4%)	11 (4.1%)	.044
Borderline abnormal	128 (4.9%)	20 (7.5%)	
Normal	2398 (91.4%)	235 (88.0%)	
**Regular antidepressant use**			
No	2223 (84.7%)	183 (68.5%)	<.001
Yes	396 (15.1%)	82 (30.7%)	

^*^Wilcoxon rank sum test; Fisher’s exact test

^†^Relative geographic remoteness in Australia is measured by calculating road distance from various populated locations.

**Table 4:** Demographic and clinical data of consenters versus nonconsenters

	**Nonconsenters** *N* = 180^*^	**Consenters** *N* = 87^*^	***p*** ^†^
**Age (in years)**			.97
Mean (SD)	66 (8)	66 (7)	
Median [Min, Max]	65 [53, 89]	66 [53, 90]	
Missing	3	0	
**Sex**			.67
Female	100 (56%)	45 (52%)	
Male	76 (43%)	42 (48%)	
Other	1 (0.6%)	0 (0%)	
Missing	3	0	
**Marital status**			.76
Married	116 (65%)	59 (68%)	
Separated or divorced	22 (12%)	6 (6.9%)	
Defacto	18 (10%)	9 (10%)	
Single	13 (7.3%)	9 (10%)	
Widowed	7 (3.9%)	4 (4.6%)	
Other	2 (1.1%)	0 (0%)	
Prefer not to say	1 (0.6%)	0 (0%)	
Missing	1	0	
**Highest level of education**			.28
Primary School	0 (0%)	0 (0%)	
High School	27 (15%)	8 (9.2%)	
Other	2 (1.1%)	0 (0%)	
Diploma/Associate Degree	39 (22%)	14 (16%)	
Certificate or Apprenticeship (including Cert 2, 3, or 4)	26 (15%)	10 (11%)	
Bachelor’s Degree	29 (16%)	18 (21%)	
Higher University degree (Honors, Graduate Diploma, Master’s or PhD)	56 (31%)	37 (43%)	
Missing	1	0	
**Years of education**			.22
Mean (SD)	15.2 (4.8)	15.8 (5.6)	
Median [Min, Max]	15.0 [0.0, 25.0]	17.0 [0.0, 35.0]	
**Currently employed**			.89
No	106 (59%)	52 (60%)	
Yes	74 (41%)	35 (40%)	
**Currently retired**			.93
N/A	1 (0.6%)	0 (0%)	
No	65 (39%)	32 (37%)	
Yes	102 (61%)	54 (63%)	
Missing	12	1	
**Number of children**			.15
Mean (SD)	2.49 (1.74)	2.18 (2.12)	
Median [Min, Max]	2.00 [0.00, 9.00]	2.00 [0.00, 13.00]	
Missing	93	30	
**Remoteness area**			.95
Inner Regional Australia	130 (74%)	64 (74%)	
Outer Regional Australia	45 (26%)	21 (24%)	
Remote Australia	1 (0.6%)	1 (1.2%)	
Unknown	4	1	
**Country of birth**			.42
Australia	100 (75%)	56 (72%)	
United Kingdom	18 (13%)	8 (10%)	
United States	4 (3%)	5 (6%)	
New Zealand	2 (1.5%)	1 (1.3%)	
Other	8 (6%)	8 (10%)	
Netherlands	1 (0.7%)	1 (1.3%)	
Papua New Guinea	1 (0.7%)	1 (1.3%)	
South Africa	0 (0%)	1 (1.3%)	
Unknown	46	9	
**Aboriginal and/or Torres Strait Islander origin**			.50
No	128 (96%)	72 (94%)	
Yes, Aboriginal	5 (3.8%)	5 (6.5%)	
Unknown	47	10	
**Sleep quality**			.18
Good Sleep Quality	61 (34%)	37 (43%)	
Poor Sleep Quality	118 (66%)	50 (57%)	
Unknown	1	0	
**Anxiety Level**			.21
Abnormal	27 (15%)	8 (9.2%)	
Borderline abnormal	26 (15%)	9 (10%)	
Normal	126 (70%)	70 (80%)	
Unknown	1	0	
**Depression level**			0.31
Abnormal	8 (4.5%)	1 (1.1%)	
Borderline abnormal	22 (12%)	8 (9.2%)	
Normal	149 (83%)	78 (90%)	
Unknown	1	0	
**Regular antidepressant use**			.19
No	119 (66%)	64 (74%)	
Yes	60 (34%)	22 (26%)	
Unknown	1	1	

^*^ *n* (%).

^†^Wilcoxon rank sum test; Fisher’s exact test; Pearson’s chi-squared test.

**Figure 3:**
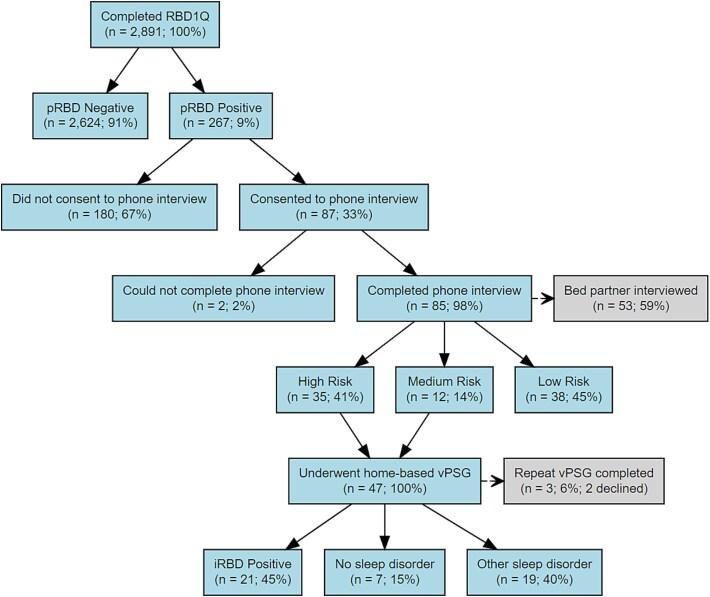
Flowchart of detection protocol. Graphical representation of the TASLON detection protocol preliminary results, from screening to confirmed iRBD.

**Figure 4:**
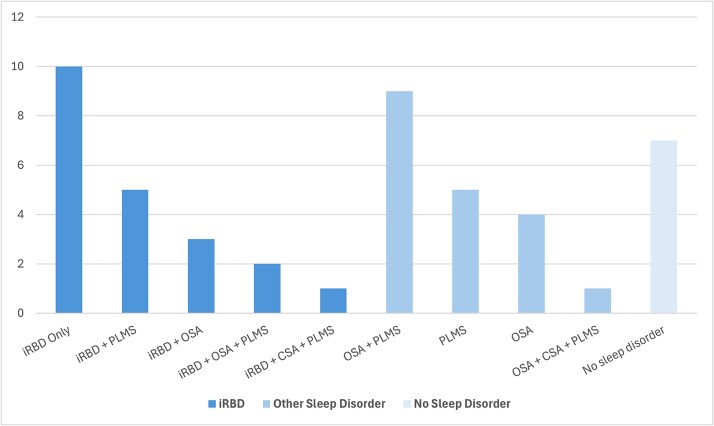
Sleep disorders detected through home-based vPSG. Abbreviations: iRBD, isolated REM sleep behavior disorder; OSA, obstructive sleep apnea; PLMS, periodic leg movements in sleep; CSA, central sleep apnea.

Of the 267 who screened positive for pRBD, 87 responded to the project invitation and consented to be contacted to complete the TASLON screening interview. Demographics of consenters versus nonconsenters can be seen in Table 4. Fifty-three bed partners also consented to contribute to the interview. Two participants were noncontactable or unable to complete the phone call. A total of 35 participants were determined to be high-risk for iRBD, 12 were medium-risk, and 38 were low-risk. At the time of publication, a total of 47 participants from the high- and medium-risk groups have undergone a home-based vPSG (2 completed in a hotel), and 21 were found to show evidence of iRBD (see Figure 3). Of these, 3 had a pre-existing diagnosis of OSA and used CPAP therapy during their vPSG. Six were found to have comorbid OSA or central sleep apnea (CSA), and 8 showed evidence of periodic leg movements in sleep (PLMS) (see Figure 4).

Seven participants did not show any evidence of a sleep disorder, and the remaining participants showed evidence of either OSA, CSA, PLMS, or a combination of these (see Figure 4). Five vPSGs were not interpretable due to either EEG or EMG lead loss/misplacement, poor impedance, or technical failure of the recording, and participants were invited to undertake a repeat study. Three participants consented to a repeat, one declined, and one relocated interstate and was unable to continue participation in the project. 81% (17/21) of confirmed iRBD participants had bed partner responses at the telephone screen stage, as compared to 56% (14/26) of iRBD negative participants. Demographic and clinical data of those with and without iRBD can be seen in Table 5, along with vPSG data from each group in Table 6. No significant differences were found between the groups on any of these variables, including regular antidepressant use.

**Table 5:** Demographic and clinical characteristics of the confirmed iRBD group versus the negative iRBD group

	**iRBD confirmed** **(*N* = 21)**	**iRBD** **negative** **(*N* = 26)**	*p* ^*^
**Age (in years)**			
Mean (SD)	68 (7)	67 (8)	.84
Median [Min, Max]	69 [55, 82]	68 [53, 79]	
**Sex**			
Female	11 (52%)	11 (42%)	.49
Male	10 (48%)	15 (58%)	
**Marital status**			
Married	17 (81%)	13 (50%)	
Defacto	2 (9.5%)	3 (12%)	.11
Single	2 (9.5%)	4 (15%)	
Separated or divorced	0 (0%)	5 (19%)	
Widowed	0 (0%)	1 (3.8%)	
**Highest level of education**			
Bachelor’s Degree	6 (29%)	2 (23%)	.46
Certificate or Apprenticeship (including Cert 2, 3 or 4)	2 (9.5%)	3 (12%)	
Diploma/Associate Degree	5 (24%)	6 (23%)	
High School	0 (0%)	4 (15%)	
Higher University degree (Honors, Graduate Diploma, Master’s or PhD)	8 (38%)	7 (27%)	
**Currently employment**			
No	14 (67%)	19 (73%)	.63
Yes	7 (33%)	7 (27%)	
**Currently retired**			
No	5 (24%)	7 (28%)	.75
Yes	16 (76%)	18 (72%)	
Unknown	0 (0%)	1	
**Number of children**			
Mean (SD)	2.14 (1.29)	2.58 (3.06)	.93
Median [Min, Max]	2.50 [0.00, 4.00]	2.00 [0.00, 13.00]	
Unknown	7	7	
**Remoteness area** ^†^			
Inner Regional Australia	17 (81%)	22 (88%)	.69
Outer Regional Australia	4 (19%)	3 (12%)	
Unknown	0	1	
**Country/Region of birth**			
Australia and New Zealand	13 (67%)	19 (83%)	.06
United Kingdom and Europe	3 (11%)	0 (0%)	
Africa	1 (5.6%)	1 (4.3%)	
United States	0 (0%)	2 (8.7%)	
Asia	1 (5.6%)	1 (4.3)	
Unknown	3	3	
**Aboriginal and/or Torres Strait Islander origin**			
No	17 (100%)	20 (87%)	.25
Yes, Aboriginal	0 (0%)	3 (13%)	
Unknown	4	3	
**Sleep quality**			
Good Sleep Quality	9 (43%)	8 (31%)	.39
Poor Sleep Quality	12 (57%)	18 (69%)	
**Anxiety level**			
Abnormal	2 (9.5%)	2 (7.7%)	.85
Borderline abnormal	1 (4.8%)	3 (12%)	
Normal	18 (86%)	21 (81%)	
**Depression level**			
Abnormal	0 (0%)	0 (%)	.36
Borderline abnormal	1 (4.8%)	4 (15%)	
Normal	20 (95%)	22 (85%)	
**Regular antidepressant use**			
No	14 (67%)	15 (60%)	.64
Yes	7 (33%)	10 (40%)	
Unknown	0	1	

^*^Wilcoxon rank sum test; Pearson’s chi-squared test; Fisher’s exact test.

^†^Relative geographic remoteness in Australia is measured by calculating road distance from various populated locations.

**Table 6:** Polysomnographic data between the iRBD Positive and negative groups

	**Negative** **(*N* = 26)**	**Positive** **(*N* = 21)**	***p***
**Total sleep time (min)**			
Mean (SD)	381 (66.2)	428 (81.3)	.0653
Median [Min, Max]	392 [204, 487]	436 [249, 571]	
**Wake after sleep onset (min)**			
Mean (SD)	84.2 (44.4)	108 (66.5)	.165
Median [Min, Max]	69.5 [18.5, 175]	93.0 [10.0, 292]	
**Sleep efficiency (%)**			
Mean (SD)	76.7 (12.8)	75.6 (13.6)	.657
Median [Min, Max]	78.3 [35.8, 91.8]	77.1 [43.2, 97.4]	
**Sleep onset latency (min)**			
Mean (SD)	23.2 (43.3)	22.1 (19.1)	.674
Median [Min, Max]	14.3 [1.50, 219]	14.5 [1.50, 68.5]	
**REM latency (min)**			
Mean (SD)	127 (92.9)	165 (129)	.222
Median [Min, Max]	89.5 [13.5, 356]	120 [43.0, 581]	
**Stage N1 (%)**			
Mean (SD)	10.0 (5.30)	9.91 (5.67)	.973
Median [Min, Max]	9.15 [1.40, 19.9]	9.20 [2.10, 23.3]	
**Stage N2 (%)**			
Mean (SD)	54.1 (13.0)	52.5 (12.4)	.716
Median [Min, Max]	53.2 [31.6, 79.9]	50.4 [29.6, 75.3]	
**Stage N3 (%)**			
Mean (SD)	17.4 (12.2)	16.7 (10.3)	.882
Median [Min, Max]	14.3 [4.10, 50.9]	14.4 [0, 37.2]	
**Stage REM (%)**			
Mean (SD)	18.6 (7.40)	20.8 (6.72)	.481
Median [Min, Max]	19.7 [0, 30.8]	19.9 [10.7, 39.9]	
**PLMS Index (/h)**			
Mean (SD)	28.4 (30.1)	37.2 (46.9)	.873
Median [Min, Max]	18.5 [0, 125]	30.9 [0, 170]	
**AHI (/h)**			
Mean (SD)	4.90 (6.43)	6.08 (10.3)	.792
Median [Min, Max]	2.30 [0, 22.5]	1.50 [0, 38.5]	
**O**_**2**_ **saturation below 90% (min)**			
Mean (SD)	25.3 (42.6)	32.0 (43.1)	.485
Median [Min, Max]	3.00 [0, 157]	15.0 [0, 151]	

**Figure 5:**
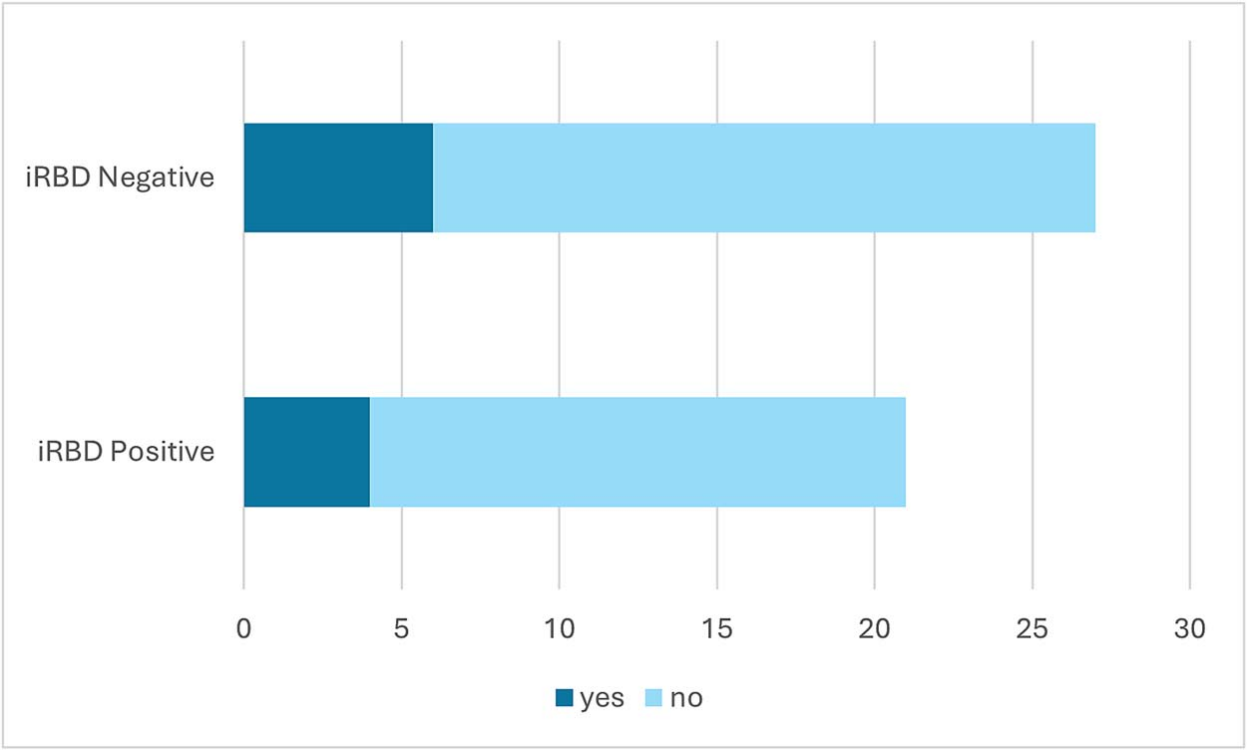
Bar chart showing the number of participants in each group who had discussed their RBD symptoms with a doctor prior to involvement in the project.

Analysis of telephone screening data revealed that only 21% (10/47) of participants had discussed their symptoms with a doctor prior to participating in the study (see Figure 5). One participant was referred to a specialist for evaluation and based on history alone, was diagnosed with probable RBD. Reported responses to these discussions with a doctor included: diagnoses of stress or restless legs syndrome; changing medications to see if symptoms may be a side effect; or reassurances that the symptoms were normal and should be dismissed.

**Figure 6:**
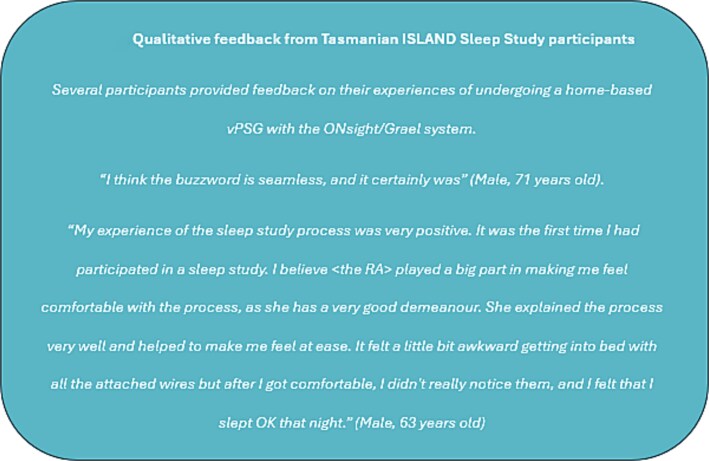
Qualitative feedback from Tasmanian ISLAND Sleep Study participants. Several participants provided feedback on their experiences of undergoing a home-based vPSG with the ONsight/Grael system.

Twenty-four participants also completed a usability questionnaire and provided qualitative feedback regarding their home-based vPSG experience; a sample of this can be seen in Figure 6. One hundred percent of participants agreed that they felt comfortable participating in the screening call and explaining their sleep symptoms to the researcher. When asked if they felt comfortable wearing the sleep study sensors, 95% agreed. The majority, 91%, agreed that they would prefer to have a home-based sleep study rather than a hospital-based sleep study, and 77% agreed that they gained insights about their sleep by participating in this research project (see Supplementary Table S2 for full results).

## Discussion

Preliminary findings from the Tasmanian ISLAND Sleep Study show that the TASLON three-step approach to identifying iRBD in the community—remote from the research center—is feasible and acceptable. This is a vital step to improving diagnosis and care in this population of older adults who are likely in the earliest stages of alpha-synuclein-related disease. Only one participant had received prior medical advice regarding iRBD-related symptoms before participation in this study, showing that there is a significant under-recognition of iRBD in the community and amongst medical professionals. So far, the use of a semi-structured clinical interview in combination with home-based vPSG has identified 21 cases of iRBD (and mimics in 19 cases) in Tasmania, Australia, from a pool of 87 who agreed to be interviewed for this study. This demonstrates that our approach has strong potential predictive value, which will be assessed in future work from this group. We utilized in-depth clinical knowledge from clinicians and researchers who work closely with iRBD populations in the development of this semi-structured interview and ensured that a wide range of relevant questions were included. It is envisaged that this interview can be used in both research and clinical environments to accurately identify those at highest risk of having iRBD. We also took into account important considerations around risk disclosure in iRBD when designing the study, such as carefully eliciting participants’ wish for disclosure, preserving their autonomy, and providing appropriate information [43]. We have previously undertaken consumer feedback and co-design with adult cohorts in Tasmania, through in-person groups and questionnaires: these have expressed a desire to be informed of increased risks of NDD so there is an opportunity to maximize brain health [44]. This aligns with other studies of patient perspectives on iRBD risk disclosure [45], but we recognize it remains unclear if these findings generalize to non-research cohorts, and there are gaps around what level of information people with iRBD prefer [45, 46]. However, without any known neuroprotective therapies available, it would seem pragmatic to extend the same brain health principles in this “at-risk” group, through lifestyle and medical risk modification, which are known to reduce dementia risk [19]. Comprehensive follow-up was conscientiously provided for participants diagnosed with iRBD in this research study, ensuring that safety and risk awareness are key components of the research and diagnostic process. This is a longitudinal study, and participants will be invited for specialist follow-up and support for its duration, including obtaining participant perspectives on this post-diagnostic process, so we can continue to evolve the protocols and consider person-centered design and preferences into the future.

### Evidence for community-based screening

Several community screening approaches for iRBD have previously been published [47–49], and a comparable three-step method for iRBD detection has recently been used in Germany, finding similar results [50]. Seger et al. recruited participants by advertising the RBD1Q in local newspapers, followed by a structured telephone screening consisting of several validated questionnaires to cover a broad range of sleep disturbances, before selecting certain participants to undergo vPSG. Their method detected 185 participants to undergo telephone screening, followed by 124 vPSGs (at home or in a hotel room) based upon expert evaluation of questionnaire answers. This study found that 62% of participants who had a vPSG (78/124) had iRBD, which is slightly higher than our findings of 45% (21/47). The proportion of females was higher in the current study compared to Seger et al.’s, with 55% females compared to 20%. This resulted in a near equal sex split in the positive and negative iRBD groups in this study; 52% and 42% respectively, whereas Seger et al had a lower number of females in both their positive and negative groups (28% and 14% respectively). Such findings suggest the importance of including a greater proportion of females in iRBD research to better understand potential sex differences within iRBD. Future work will build upon the efficacy of the interview used in this study by analyzing a greater number of participant responses and vPSG results in depth, using regression and receiver operating characteristic analyses to determine the positive predictive value of the clinical interview to detect iRBD compared to the gold-standard in-laboratory vPSG.

### Feasibility of current diagnostic requirements for iRBD

Presently, gold-standard PSG assessment for iRBD requires an overnight stay in a hospital or sleep laboratory whilst being attended by a sleep technician, scientist, or physiologist. The PSG required to detect iRBD is also more complex than standard PSG, as the current AASM manual for the scoring of sleep and associated events [6] recommends the use of additional sensors on upper limbs, which are not routinely included in a standard PSG. Not only is access to these services limited geographically, but they may also be costly, with fees reaching up to USD 2000 for a standard PSG admission [51]. Wait times vary between countries but can be 12 months or more for those awaiting testing for common sleep disorders, such as OSA [52], and are likely to be longer for those in need of the iRBD-specific PSG. Additionally, travel to and from facilities can also pose a significant challenge for those with added comorbidities. In fact, one study from North America found that the average delay in diagnosis for people suspected of RBD was nine years [5]. Researchers interested in iRBD recognize that reliable, ambulatory PSGs are urgently needed to detect iRBD within the home and the community in order to speed up accurate iRBD diagnosis, and to facilitate earlier treatment and recruitment into clinical trials.

### Home-based assessment may be the solution

Home-based PSG is favored over laboratory or hospital-based PSG for most individuals, as it is more convenient, comfortable, and affordable [53–56]. To our knowledge, this is one of the first studies to implement a home-based vPSG protocol for the detection of iRBD. The Compumedics ONsight A.V.S system, in combination with the Grael 4K amplifier, allows for the collection of the exact same data that would be captured in a laboratory or hospital-based vPSG, but at home. Not only does this allow for the precise and accurate physiological data collection needed for iRBD detection, but it also ensures greater participant comfort and encourages better sleep quality and increased total sleep time. Whilst technical assistance was available overnight if needed, we found that participants did not require this, and that the home-based vPSG system was acceptable and user-friendly, suggesting ease of replicability in the future.

### Limitations of home-based vPSG and the TASLON protocol

Unfortunately, unattended vPSG does pose a risk of failure due to the potential loss or displacement of body sensors overnight, which would normally be replaced during a lab or hospital-based vPSG by the attending technician. Our current limited data suggest a failure rate of 10% for this study, which is in line with previous home-based PSG studies for OSA estimating failure rates between 4% and 20% [57]. This is also a particular risk for people suspected of having iRBD, as dream-related movements make it more likely for sensors to become displaced on the body or dislodged from the headbox. Of the two participants who have undergone a repeat study, both were confirmed to have iRBD on their second attempt. One way to mitigate this limitation is the inclusion of video recording, which has the potential to provide additional data if EMG signals are lost. For example, if leg or arm EMG signal is lost due to motor activity, but EEG is retained, sleep physiologists and specialists may still be able to determine that a dream enactment episode is evident if REM sleep is observed alongside visualization of limb movements.

Currently, the ICSD-3-TR criteria for iRBD diagnoses do not require video-recorded evidence of iRBD, although the use of synchronized video recording during PSG is recommended by the AASM, and the International RBD Study Group [58] as a necessary addition. Nevertheless, interpretation of visual data depends on the scrutiny of the reviewer and would not be reliable if both vital EEG and EMG signals were lost. Another option could be to show bed partners the vPSG set-up whilst it is underway and then guide them on how to reattach or replace sensors if they are found to be lost overnight, though this would not be possible for people sleeping on their own. A further limitation to unattended home-based vPSG in this study is the use of a newly configured system, which has not been validated against a gold-standard laboratory-based system. Even though the system collects all required data points for RBD detection, a small risk for incorrect detection remains. Technical troubleshooting was required by researchers and technical support staff whilst the equipment was in use in the field, which caused a delay in data collection at some points throughout the project. The size and number of cases needed to transport the vPSG equipment from home to home is also large and cumbersome, particularly for one person to manage. Future research into failure rate improvements would benefit from better connectivity of sensor leads to the headbox, strengthened sensor adhesive, appropriate calibrations, and thorough testing of equipment prior to data collection. Improved design approaches to minimize bulk and maximize portability of vPSG equipment would further enhance the research experience for both researchers and participants in the future.

An additional limitation of this study is the omission of a control group from the full protocol. Although the exclusion of controls in the vPSG cohort may be considered an oversight since negative predictive value (NPV) cannot be calculated, it must be considered that the lower bound of NPV is inversely proportional to disease prevalence [59]. Put simply, even a test with poor sensitivity cannot miss more cases than exist. It follows that, where prevalence is low, the practical value of empirically estimated NPV is limited, provided the screening test is sufficiently sensitive. However, consideration should be given to the inclusion of controls in future studies in populations where we might expect the prevalence of iRBD to be higher, particularly where the cost of a missed diagnosis to the patient may be steep.

One final limitation to be acknowledged is the cohort of the ISLAND Sleep Study in general. Tasmania has the oldest demographics of all Australian states with a median age of 42 compared Australia’s median age of 38 [60]. It also comprises over 220 000 adults aged 50 and over within its total population of 550 000 [61], making Tasmania an ideal location for research into age-related diseases. However, the ISLAND cohort is 71% female compared to 51% of the overall Tasmanian population, and while 50% of the ISLAND sample have completed a Bachelor’s degree or higher, this is only 21% in the wider population [61, 62]. In addition, the cohort is computer literate, has access to the internet, and is known to be motivated to learn about health risks and maximize brain health, which is not comparable to the general population of Tasmania. These disparities mean that preliminary results from this study will need to be replicated in more representative populations in the future.

## Conclusion

In conclusion, these preliminary data show that the 3-step approach using the TASLON iRBD screening interview is useful for the assessment of people suspected of having iRBD. Unfortunately, iRBD is difficult to diagnose, as overnight movements can be misconstrued for other, more common sleep disorders. The use of a standardized semi-structured clinical interview alongside home-based vPSG would greatly improve the process of iRBD diagnoses in the community. It would streamline the path to vPSG by identifying those at highest risk of iRBD, thus improving access to diagnostic testing for those who otherwise may not have been identified.

## Supplementary Material

Supplementary_Tables_zsaf313

## Data Availability

Data available on request: the data underlying this article will be shared on reasonable request to the corresponding author.
