# Supplementary material for: The Tasmania-London protocol to detect isolated rapid eye movement sleep behavior disorder using home-based video-polysomnography
Source: Sleep. 2025 Oct 7;49(3):zsaf313. doi: 10.1093/sleep/zsaf313 (PMC13017356; doi:10.1093/sleep/zsaf313)
Supplement: Supplementary_Tables_zsaf313 [file supplementary_tables_zsaf313.docx]

**The Tasmania-London (TASLON) protocol to detect isolated REM sleep behaviour disorder using home-based video-polysomnography.**

Samantha Bramich^1^, Alastair J Noyce^1,2^, Anna E King^1^, Séan Higgins^3^, Cristina Simonet^2^, Aidan Bindoff^1^, Sharon L Naismith^4^, James C Vickers^1^, Laura Pérez-Carbonell^2,3,5*^ and Jane Alty^1,6,7*^

*Joint senior author

1. Wicking Dementia Research and Education Centre, University of Tasmania, Australia
2. Centre for Preventive Neurology, Wolfson Institute of Population Health, Queen Mary University of London, United Kingdom
3. Sleep Disorders Centre, Guy's and St Thomas' NHS Foundation Trust, London, United Kingdom
4. School of Psychology, Brain and Mind Centre, University of Sydney, Australia
5. Institute of Psychiatry, Psychology and Neuroscience, King’s College London, United Kingdom
6. School of Medicine, University of Tasmania, Australia
7. Neurology Department, Royal Hobart Hospital, Tasmania, Australia

Corresponding authors:
Samantha Bramich and Professor Jane Alty
samantha.bramich@utas.edu.au & jane.alty@utas.edu.au
+61403276742
University of Tasmania, 17 Liverpool Street,
Hobart, Tasmania Australia 7000

***Supplementary Table 1.* Online Health and Sleep Questionnaires**

| **Questionnaire** | **Assessment Domain** |
| --- | --- |
| The Mannheim Dream questionnaire (MADRE) (36) | Dream history and characteristics |
| The REM Sleep Behaviour Disorder Single Question Screen (RBD1Q) (29) | RBD screening |
| REM Sleep Behaviour Disorder Screening Questionnaire (RBDSQ) (28) | RBD screening |
| Pittsburgh Sleep Quality Index (PSQI) (31) | Sleep quality |
| STOP-BANG Sleep Apnoea Questionnaire (32) | Sleep apnoea |
| The Composite Autonomic Symptom Score (COMPASS-31) (33) | Autonomic dysfunction |
| Michael J Fox Parkinson’s Screening Questionnaire (34) | Parkinson’s motor symptoms |
| COVID-19 | Infection acquired and vaccination status |
| Short Form-McGill Pain Questionnaire-2 (SF-MPQ-2) (35) | Pain |

**Supplementary Table 2. Home-sleep study usability questionnaire results.**

| **Question** | **Strongly Agree** | **Moderately Agree** | **Agree** | **Neutral** | **Disagree** | **Moderately Disagree** | **Strongly Disagree** |
| --- | --- | --- | --- | --- | --- | --- | --- |
| I felt comfortable participating in the screening call and explaining my sleep symptoms to the researcher | 16 (73%) | 0 (0%) | 6 (27%) | 0 (0%) | 0 (0%) | 0 (0%) | 0 (0%) |
| I found the sleep study set up attachment of sensors to be a well organised process | 14 (64%) | 0 (0%) | 8 (36%) | 0 (0%) | 0 (0%) | 0 (0%) | 0 (0%) |
| I felt comfortable wearing the sleep study sensors | 4 (18%) | 7 (32%) | 10 (45%) | 1 (4.5%) | 0 (0%) | 0 (0%) | 0 (0%) |
| It was easy to go about my usual evening activities walking eating toileting etc. with the sleep study sensors attached | 3 (14%) | 11 (50%) | 5 (23%) | 2 (9.1%) | 0 (0%) | 1 (4.5%) | 0 (0%) |
| I slept well with the sleep study sensors attached overnight | 5 (23%) | 7 (32%) | 6 (27%) | 2 (9.1%) | 0 (0%) | 2 (9.1%) | 0 (0%) |
| I would prefer to have a home based sleep study rather than a hospital based sleep study | 12 (55%) | 2 (9.1%) | 6 (27%) | 0 (0%) | 1 (4.5%) | 0 (0%) | 1 (4.5%) |
| I gained insights about my sleep by participating in the sleep study project | 10 (45%) | 2 (9.1%) | 5 (23%) | 4 (18%) | 0 (0%) | 0 (0%) | 1 (4.5%) |
|  | | | | | | | |
